# Urokinase-type Plasminogen Activator Promotes Synaptic Recovery in the Ischemic Brain

**Published:** 2018-03-20

**Authors:** Manuel Yepes

**Affiliations:** 1 Division of Neuropharmacology and Neurologic Diseases, Yerkes National Primate Research Center, Atlanta, GA, USA; 2 Department of Neurology, Center for Neurodegenerative Disease, Emory University School of Medicine, Atlanta, GA, USA; 3 Department of Neurology, Veterans Affairs Medical Center, Atlanta, GA, USA

## Abstract

Synaptic dysfunction underlies the development of neurological impairment following an acute ischemic stroke. Unfortunately, to this date there is no therapeutic approach to protect and repair the synapse that has suffered an ischemic injury. However, recent research with *in vitro* models of hypoxia, *in vivo* models of cerebral ischemia and different neuroradiological techniques has revealed that during the recovery phase from a hypoxic injury neurons release the serine proteinase urokinase-type plasminogen activator (uPA) and astrocytes recruit its receptor (uPAR) to their plasma membrane; and that binding of neuronal uPA to astrocytic uPAR promotes the recovery of the “tripartite synapse” that has suffered an acute ischemic injury. The translational relevance of these findings is underscored by the fact that intravenous treatment with recombinant uPA promotes synaptic recovery and functional improvement following an acute ischemic stroke.

## Introduction

Ischemic stroke is the second cause of mortality in the world and the 5^th^ in USA [1]. Fortunately, the development of new medical and neuroradiological strategies to treat acute ischemic stroke patients has led to a sustained decreased in stroke mortality [2]. However, this has also resulted in a significant increase in the number of patients that survive an ischemic stroke with different degrees of disability. Unfortunately, in contrast with the treatment of acute ischemic stroke patients, to this date there is no effective therapeutic strategy to promote neurological recovery among ischemic stroke survivors.

Despite the fact that neurorepair in the ischemic brain is a very complex process, there is a growing consensus around the concept that synaptic dysfunction underlies the development of functional impairment after an ischemic stroke [3]. Indeed, it is estimated that one minute of ischemia in the human brain destroys 14 billion synapses [4]. Thus, protection of synapses that are suffering an ischemic injury and repair of those that have survived an acute ischemic stroke, are the cornerstone for any therapy aimed to promote neurological recovery among ischemic stroke patients.

## The Plasminogen Activating System

The plasminogen activation (PA) system is an enzymatic cascade that promotes the degradation of fibrin by catalyzing the conversion of plasminogen into the broad-spectrum protease plasmin upon its activation by two serine proteinases: tissue-type plasminogen activator (tPA) and urokinase-type plasminogen activator (uPA) [5]. Thus, based on their ability to generate plasmin, thrombolysis with either recombinant uPA [6] or tPA [7] has shown to promote neurological improvement when administered to acute ischemic stroke patients within few hours of onset of symptoms. Remarkably, soon thereafter became evident that besides their fibrinolytic role, uPA and tPA also play a central role in the central nervous system. Accordingly, it was shown that neuronal activity induces the release of tPA in the synapse, where it mediates the development of neuronal plasticity [8], learning [9,10], stress-induced anxiety [11], and visual cortex plasticity [12]. In contrast, it was considered that uPA plays a central role during development but not in the mature brain [13].

### tPA and uPA in the ischemic brain

A substantial amount of experimental data indicates that the activity of uPA and tPA increase in the ischemic brain. However, while tPA expression increases within minutes of the ischemic injury and decreases again to baseline levels in less than 3 hours, uPA activity raises only 6–24 hours after the end of the acute ischemic injury [14,15]. Interestingly, whereas this finding originated a large number of papers on the role of tPA in the ischemic brain, very few research groups focused their work on the effect of uPA. Indeed, since then it has been discovered that the release of tPA in the ischemic brain promotes synaptic protection [16,17], and regulates the permeability of the blood-brain barrier [14], our knowledge of the role of uPA is very limited.

### Urokinase-type plasminogen activator

uPA is a serine proteinase that catalyzes the conversion of plasminogen into plasmin on the cell surface [18]. It is produced as a single-chain inactive protein that is cleaved into two-chain active uPA assembled by an amino-terminal growth factor, a kringle, and a carboxy-terminal catalytic domain. Its name was derived from the observation that the renal tubular epithelium seems to be its most abundant source *in vivo*. However, subsequent studies found that wound repair and angiogenesis also induce the expression of uPA in other tissues [19]. uPA binds to a three-domain receptor (uPAR) attached to the plasma membrane through a glycosyl phosphatidylinositol (GPI) anchor [20]. Upon binding to uPAR, activated uPA (and inactive uPA) cleaves the zymogen plasminogen generating plasmin which reciprocally cleaves and activates pro-uPA [21]. The activity of the uPA/uPAR system is regulated by plasminogen activator inhibitor-1 (PAI-1), which induces the internalization and degradation of uPAR-bound uPA via de low-density lipoprotein receptor-related protein-1 (LRP-1). Then internalized uPAR and LRP1 recycle back to the cell surface [20].

### Urokinase in the ischemic brain

Despite the fact that for a long time it was believed that uPA/ uPAR binding in the brain is important only during development [13], recent studies revealed the intriguing finding that neurons but not astrocytes release uPA while recovering from an episode of hypoxia *in vitro* and cerebral ischemia *in vivo*. Furthermore, these studies also showed that astrocytes do not release uPA, but instead that they recruit uPAR to their plasma membrane in response to the hypoxic injury. Interestingly, genetic deficiency of neither uPA nor uPA has an effect on neuronal death in *in vitro* models of hypoxia and *in vivo* models of cerebral ischemia [22]. In contrast, compared to their wild-type controls, animals genetically deficient on either uPA (uPA^−/−^) or uPAR (uPAR^−/−^) have impaired functional recovery following an acute ischemic injury [23]. More importantly, intravenous treatment with recombinant uPA (ruPA) after the onset of the ischemic injury, at doses similar to those used in acute ischemic stroke patients [6] induced neurological improvement in Wt and uPA^−/−^ but not uPAR^−/−^ animals. Together, these data set up the stage to develop the concept that binding of uPA to uPAR promotes neurological recovery following an acute ischemic stroke.

### Urokinase and the ischemic synapse

In an attempt to understand the mechanism whereby uPA binding to uPAR promotes neurological recovery without reducing the volume of the ischemic lesion, the brain of Wt, uPA^−/−^, and uPAR^−/−^ mice was examined with diffusion tensor imaging (DTI) following the induction of experimental cerebral ischemia. Surprisingly, it was found that compared to Wt animals, uPA^−/−^ and uPAR^−/−^ mice have increased mean diffusivity of water (MD) and decreased fractional anisotropy (Fa) in a thin area of tissue surrounding the necrotic core [22], where previous studies have shown activation of biochemical and structural changes that drive the recovery of neurons damaged by the ischemic injury [24]. These data led to propose a model in which binding of uPA released by neurons to uPAR recruited to the plasma membrane during the recovery phase from a hypoxic/ischemic injury have a protective effect on the synapse. More importantly, these findings suggest that uPA and uPAR mediate a cross-talk between the different components of the “tripartite synapse” that results in preservation of its integrity and function.

### uPA-uPAR binding mediate a Cross-talk between the different components of the “Tripartite Synapse”

The term “tripartite synapse” was proposed to conceptualize the fact that owning to their intimate contact with the synapse astrocytes can regulate synaptic function [25]. Thus, to study the role of uPA/uPAR binding on the “tripartite synapse”, several studies investigated the effect of uPA/uPAR on each of its components: axons, dendrites and astrocytes.

#### uPA and the ischemic dendrite

Dendritic spines are protrusions that receive the excitatory input in the central nervous system [26]. Importantly, several *in vivo* studies using two-photon confocal microscopy have shown that cerebral ischemia has a profound effect on their morphology associated with synaptic integrity and functional outcome. Hence, while dendritic spines located inside the necrotic core are irreversibly damaged by the ischemic injury, those located within 1 mm from its border are replaced by varicosities, and remarkably, if the cause of the ischemic injury disappears, a significant proportion of spines reemerge from these varicosities [27]. A role for uPA in the recovery of dendritic spines that have suffered an ischemia injury, was discovered by the observation that Wt, but not uPA^−/−^, or uPAR^−/−^ mice, or animals in which a 4 amino acids mutation in endogenous uPA precludes its binding to endogenous uPAR (Plau^GFDhu/GFDhu^) [28] exhibit dendritic spine recovery after an ischemic injury. Furthermore, intravenous treatment with recombinant uPA was shown to induce dendritic spine recovery in Wt, uPA^−/−^ and Plau^GFDhu/GFDhu^ but not uPAR^−/−^ mice. Together, these data indicate that binding of endogenous or recombinant uPA to endogenous uPAR promotes dendritic spine recovery in the ischemic brain.

#### Effect of uPA on the presynaptic terminal

Growth cones are highly dynamic structures located at the tip of the axon, that guide their growth during development and after an injury [29]. A typical axonal growth cone is assembled by three areas: a peripheral domain that contains F-actin bundles that form filopodium and lamellipodium-like veils, a central domain that contains microtubules, and an actinomyosin-rich transition zone between the peripheral and central domains [29]. A role for uPA/uPAR binding on the response of the axon to an injury was derived from the observation that uPAR is abundantly found in growth cones that reemerge from an injured axonal mantle, and that treatment with uPA promotes the growth of new axons following a mechanical injury. Again, as described for the postsynaptic terminal, this effect is mediated by uPAR. Furthermore, it required the activity of LRP-1, but not as an endocytic receptor, but as an inductor of the recruitment of β1-integrin to the plasma membrane. More importantly, it was found that the co-receptor activity of uPAR: β1-integrin; LRP-1 induced the recovery of injured growth cones via activation of the Ras-related C3 botulinum toxin substrate 1 (Rac-1) [23].

#### uPA induces astrocytic activation

To examine the effect of uPA on the third component of the “tripartite synapse” the expression of glial fibrillary acidic protein (GFAP) was studied in astrocyttes treated with recombinant uPA. Surprisingly, it was found that uPA-treated cells have a significant increase in GFAP expression [30]. This was an important discovery because an increase in the expression of GFAP is a marker of astrocytic activation, an evolutionarily preserved process whereby astrocytes develop well-characterized structural, genetic and biochemical changes in response to their stimulation and different forms of injury [31]. To investigate whether uPA also induces astrocytic activation in the ischemic brain, the expression of GFAP was examined in the ischemic tissue of Wt, uPA^−/−^, uPAR^−/−^ and Plau^GFDhu/GFDhu^ mice following transient occlusion of the middle cerebral artery (tMCAo) and intravenous treatment with either ruPA or a comparable volume of saline solution. These experiments revealed that uPA binding to uPAR induces astrocytic activation in the ischemic brain. Further studies showed that this effect is mediated by extracellular signal-regulated kinase ½ (ERK ½)-induced phosphorylation of the signal transducer and activator of transcription3 (STAT3). Significantly, work with neuronal: astrocytic co-cultures revealed that the interaction between astrocytic STAT3 and LRP1 in the postsynaptic terminal promote synaptic recovery in neurons that are recovering from an acute hypoxic/ischemic injury [30].

## Conclusion

In conclusion, the experimental data available to this date suggest a model in which during the recovery phased from an ischemic stroke the injured synapse releases uPA and astrocytes recruit uPAR to their plasma membrane. The interaction between neuronal uPA and astrocytic uPAR triggers a sequence of events that leads to the recovery of the presynaptic compartment mediated by β1-integrin activation of Rac 1, and the protection of the postsynaptic terminal by reorganization of its actin cytoskeleton. Together, these data indicate that treatment with ruPA or induction of release of endogenous uPA is a potential strategy to promote neurological recovery among ischemic stroke survivors.
